# Characterization of the contribution of shared environmental and genetic factors to metabolic syndrome methylation heritability and familial correlations

**DOI:** 10.1186/s12863-018-0634-7

**Published:** 2018-09-17

**Authors:** Lindsay Fernández-Rhodes, Annie Green Howard, Ran Tao, Kristin L. Young, Mariaelisa Graff, Allison E. Aiello, Kari E. North, Anne E. Justice

**Affiliations:** 1 Department of Epidemiology, University of North Carolina at Chapel Hill, 137 East Franklin Street, Chapel Hill, NC 27514 USA; 2 Carolina Population Center, University of North Carolina at Chapel Hill, 136 East Franklin Street, Chapel Hill, NC 27514 USA; 3 Department of Biostatistics, University of North Carolina at Chapel Hill, Chapel Hill, 137 East Franklin Street, Chapel Hill, Chapel Hill, NC 27514 USA; 4 Department of Biostatistics, Vanderbilt University Medical Center, 2525 West End Avenue, Nashville, TN 37203 USA

**Keywords:** Epigenetic inheritance, Methylation, Heritability, Familial correlation, Metabolic syndrome

## Abstract

**Background:**

Transgenerational epigenetic inheritance has been posited as a possible contributor to the observed heritability of metabolic syndrome (MetS). Yet the extent to which estimates of epigenetic inheritance for DNA methylation sites are inflated by environmental and genetic covariance within families is still unclear. We applied current methods to quantify the environmental and genetic contributors to the observed heritability and familial correlations of four previously associated MetS methylation sites at three genes (*CPT1A*, *SOCS3* and *ABCG1*) using real data made available through the GAW20.

**Results:**

Our findings support the role of both shared environment and genetic variation in explaining the heritability of MetS and the four MetS cytosine-phosphate-guanine (CpG) sites, although the resulting heritability estimates were indistinguishable from one another. Familial correlations by type of relative pair generally followed our expectation based on relatedness, but in the case of sister and parent pairs we observed nonsignificant trends toward greater correlation than expected, as would be consistent with the role of shared environmental factors in the inflation of our estimated correlations.

**Conclusions:**

Our work provides an interesting and flexible statistical framework for testing models of epigenetic inheritance in the context of human family studies. Future work should endeavor to replicate our findings and advance these methods to more robustly describe epigenetic inheritance patterns in human populations.

## Background

Metabolic syndrome (MetS) is a widespread problem in the United States, with 35% of U.S. adults having MetS in 2012 [1]. It is often defined by having at least three of the following: increased waist circumference (≥88 cm for women or ≥ 100 cm for men), high triglycerides (≥150 mg/dL), low high-density lipoprotein cholesterol (≤40 mg/dL for men, ≤50 mg/dL for women), hypertension (> 130 mmHg systolic and/or > 85 mmHg diastolic), and elevated fasting blood glucose (≥100 mg/dL or previous diagnosis of diabetes), or reliance on medications to correct these disturbances [2]. The MetS epidemic is on the rise in much of the world with younger generations experiencing earlier onset and higher lifetime disease burden [3].

Given the heritability that remains unexplained by established genetic variants for the subcomponents of MetS, transgenerational epigenetic inheritance has been posited as a possible contributor to the observed heritability [4]. Although cytosine-phosphate-guanine (CpG) methylation may be trans-generationally inherited, it is also possible that CpG sites are mediators of the effect of inherited genetic variant(s) on gene expression, or are biomarkers for the complex patterning of social or environmental risk factors. In fact, recent work has shed light on the complexity of how environmental risk factors within populations and across generations interact with both genetic variation and transgenerational epigenetic inheritance [5]. Yet substantial ethical and methodologic challenges remain to observationally or experimentally identifying transgenerational epigenetic inheritance in humans [4].

To date, CpG methylation sites at *CPT1A*, *SOCS3*, and *ABCG1* have been associated with MetS, or its subcomponents *(CPT1A*, *ABCG1)* [6–12]. The extent to which these associations are driven by environmental or genetic mechanisms is a source of debate and is one that has great practical implications for tailoring public health prevention. One approach to understanding the underlying mechanism is the estimation of heritability or familial correlation at CpG sites, which has been done across the methylome using twin-based studies [13], extended family-based samples from multigenerational pedigrees [7, 14], and in proof-of-principle studies in animal models [4]. However, the extent to which heritability or correlations estimates are inflated by environmental and genetic covariance within families is still unclear. Thus, robust estimates of heritability, unrelated to recapitulated environmental factors or inherited genetic variation, are needed to inform our understanding of the role of epigenetic inheritance in metabolic dysfunction as well as inform the origins of current intergenerational patterning of health disparities.

We aimed to apply current methods (ie, variance component models and correlations) to quantify the environmental and genetic contributors to the observed similarity within families at four specific MetS CpG sites. To do this, we leveraged data on 1105 adults made available through the Genetic Analysis Workshop (GAW20) to estimate the heritability at CpG sites near 3 genes (*CPT1A*, *SOCS3*, and *ABCG1*), adjusting for demographic, environmental factors and genetic variation in a stepwise fashion using both fixed and random effects. Then we estimated familial correlations of methylation profiles at these CpG sites, both with and without adjustments, and across relative pair types.

## Methods

### GAW20 methylation and genotypic data

The real GAW20 methylation and genotypic data come from 188 extended families collected from Minnesota and Utah as part of the Genetics of Lipid Lowering Drugs and Diet Network (GOLDN) study [14]. Our analytic sample consisted of 1105 GOLDN participants with MetS at baseline, as defined by the criteria described above [2], and 995 adults were typed for methylome-wide DNA methylation patterns at 485,577 CpG sites using the HM450 array following bisulfite conversion (Illumina Inc., San Diego, CA, USA) of DNA from sorted CD4+ lymphocytes at visit 2. We excluded 1 individual from a monozygotic pair and 1 individual with missing smoking status from the statistical analyses, leading to a final sample of 1103 in the MetS and 993 individuals and CpG site heritability/correlation analyses (in 3682 and 3176 pairs, respectively, that were between first and fifth relatives). A subset of 716 individuals also had genotyping from the Affymetrix Genome-Wide Human Single Nucleotide Polymorphism (SNP) Array 6.0 (Affymetrix, Inc., Santa Clara, CA, USA).

### MetS methylation loci

From the literature we selected 4 CpG sites that were previously associated with MetS (eg, *CPT1A, SOCS3*), or with Type 2 diabetes, lipids, and obesity-related traits (eg *CPT1A, ABCG1*) including: cg00574958 and cg17058475 near *CPT1A* [6, 7, 9, 11, 12]; cg18181703 in *SOCS3* [10]; and cg06500161 in *ABCG1* [7–9, 12].

### Heritability analyses

We estimated the narrow sense heritability of MetS [2] and 4 CpG methylation sites using variance component models implemented in SOLAR version 6.6.2 [15]. The CpG site residuals were scaled by 25 for stability in our SOLAR models.

No fixed effect covariates were included in our crude heritability models (Model 0). Further analyses accounted for an individual’s age and sex (female, male as referent), quadratic age effects, and their interactions with sex (Model 1). In all subsequent models, environmental covariates were added into the models in the following sequence: center (Minnesota, Utah as referent; Model 2a), followed by cigarette smoking status (former, current, never as referent; Model 2b). We then screened all these demographic and environmental fixed effects, including only the effects that remained suggestively significant in the heritability models (*P* value < 0.1). Then using the fixed effects identified in the reduced model above, we added household variance components to account for siblings and half-siblings within 15 years of each other, who were the relative pair type most likely to have shared an ‘early life’ environment at some point during their childhood or adolescence (Model 3a). Separately we added a variance component for parent pairs (if an individual was in more than 1 parental pair, taking the pairing resulting in the youngest offspring), who were the relative pair type most likely to have shared ‘later life’ environmental exposures (Model 3b). Lastly, in a fourth modeling strategy that included the same fixed effects from the reduced model (Models 1 and 2), we screened at *P* value < 0.05 local cis-acting genetic variants at each locus. To select these variants, we used publicly available 1000 Genomes phase 3 CEU (Northern Europeans from Utah) reference data to query two independent sets (pairwise linkage disequilibrium r2 < 0.05, estimated in PLINK version 1.07) [16] of genetic variants: local variants (±250 kb of the CpG site[s]), and distant variants (250–500 kb) as done previously [12]. This resulted in *n* = 8, 19, and 21 local and *n* = 6, 7, and 13 distant variants screened in heritability models for CpG sites at *CPT1A, SOCS3,* and *ABCG1*, respectively.

### Familial correlations

The expected intra−/interclass correlation for each relative pair is a function of the pairs’ expected relatedness and the CpG site-specific heritability. We estimated weighted correlations using the FCORR module of the *S.A.G.E*. version 6.4 package (http://darwin.cwru.edu/sage/) within various pair types, representing a *quasi-*independent subset of the family pedigrees. We contrasted our correlations before and after creating a residual of methylation to account for the fixed effects identified in multiple reduced heritability models, and among a subset of unrelated individual pairs.

## Results

### Heritability of MetS

The prevalence of MetS at the baseline examination of GOLDN was 38.4% and its heritability was 0.47 (Standard Error, SE = 0.10; *P* value = 1E-5, *n* = 1103) in a model where fixed effects (age, age^2^, and sex; *P* value < 0.1) explained 13% of the variation in MetS. Separately, we included variance components for early life shared environment (c^2^ = 0.21, SE = 0.09, *P* value = 7E-3), or later life shared environment (c^2^ = 0.40, SE = 0.16, *P* value = 0.01). Although the addition of these terms influenced the magnitude of the heritability estimates (h^2^ = 0.32, SE = 0.13 and h^2^ = 0.52, SE = 0.12, respectively), the resulting heritability estimates did not differ significantly.

When we added fixed effects for the 4 MetS CpG sites into the model without shared environment-related variance components, two of the CpG sites (cg00574958 at *CPT1A*, cg06500161 at *ABCG1*; *P* value <7E-5) were strongly associated with MetS and another site (cg18181703 at *SOCS3*; *P* value = 0.07) was suggestively associated with MetS. Retaining these 3 sites in the polygenic model decreased the heritability estimate slightly (h^2^ = 0.43, SE = 0.12, *P* value = 2E-5) and increased the variance explained (VE) by all the fixed covariates to 18%. The addition of random effects of early life shared environment (c^2^ = 0.23, SE = 0.11, *P* value = 0.01) decreased the heritability estimate (h^2^ = 0.24, SE = 0.15, *P* value = 0.05), resulting in a nonsignificant MetS heritability estimate, whereas accounting for shared late life environment (c^2^ = 0.28, SE = 0.19, *P* value = 0.07) increased the heritability only slightly (h^2^ = 0.46, SE = 0.12, *P* value = 1E-5).

### Heritability of MetS methylation sites

**Fig. 1:**
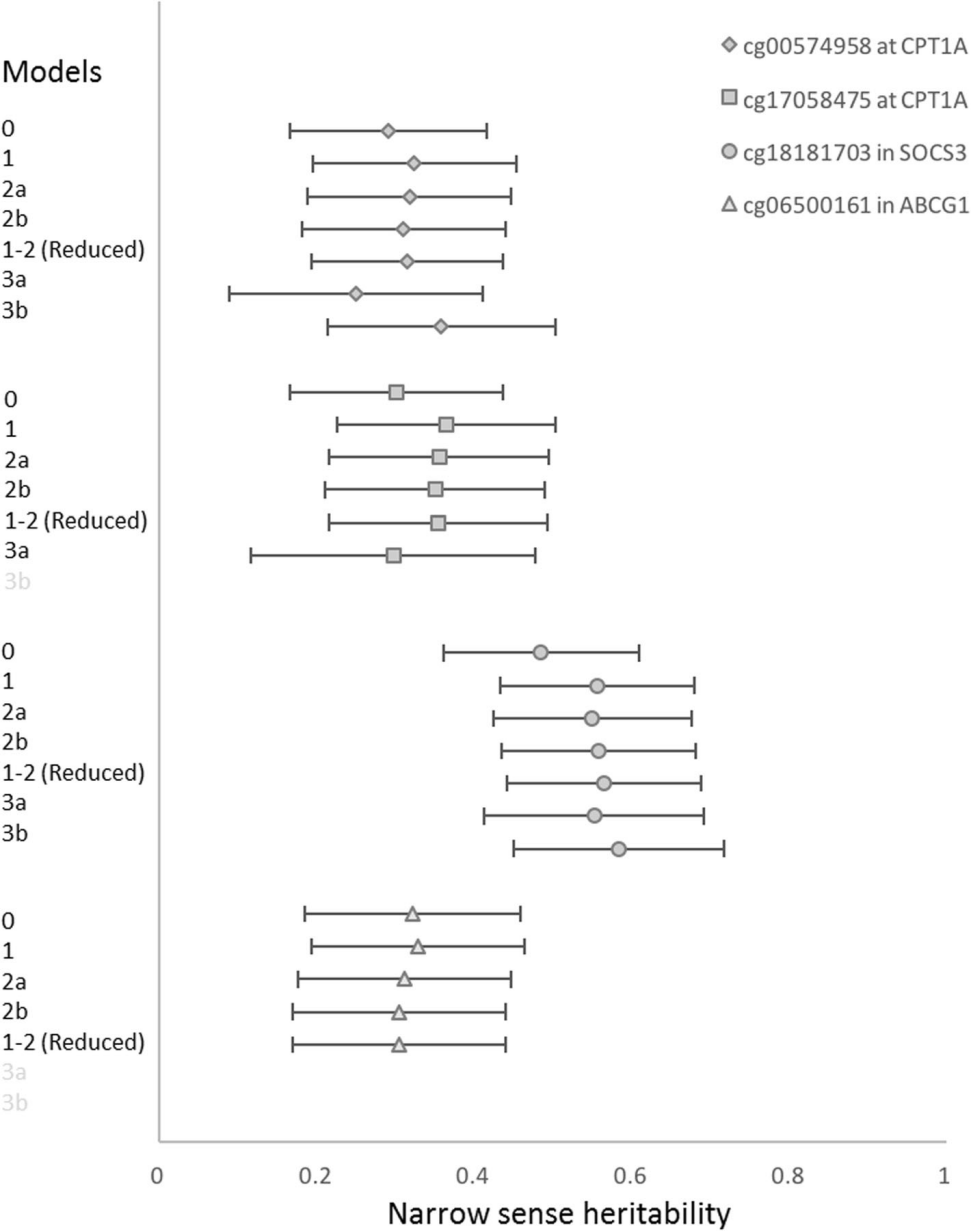
Forest plot of MetS CpG methylation heritability estimates and 95% confidence intervals among converged models (in black) that were unadjusted (Model 0) or adjusted for demographic and environmental factors (Models 1 and 2), or for shared early and late life environment (Model 3a, 3b)

The CpG site heritability estimates varied across models (Table 1), although such differences were nonsignificant (Fig. 1). The CpG site at *SOCS3* was found to be highly heritable with a value of 40% or higher in all models. Notably, when convergence was achieved heritability estimates at all CpG sites were robust to inclusion of early life and late life shared familial environments, suggesting a minimal inflation of CpG site heritability estimates resulting from these shared environments. For cg00574958 at *CPT1A,* shared early and later life variance components were both significant (Table 1).

**Table 1:** Variation in estimated additive heritability at three MetS-related CpG methylation loci adjusted (Model 0) and across increasing adjustments for demographic and environmental factors (Models 1–2), in a reduced model of fixed effects, and after including to this reduced model separate variance components for shared early life (Model 3a) and late life environment (Model 3b) in 993 participants from 188 families with all covariates and methylation information at visit 2 in the GOLDN study

Model^a^	Log Likelihood	h^2^ (c^2^)	SE of h^2^ (c^2^)	*P value* of h^2^ (c^2^)	Prop Var Exp by Cov
**cg00574958 at** ***CPT1A***					
0	− 560.399	0.292	0.064	2E-7	–
1	− 513.830	0.325	0.066	2E-8	0.085
2a	− 511.765	0.319	0.066	4E-8	0.089
2b	−509.989	0.311	0.066	1E-7	0.094
1–2 (Reduced)	−511.893	0.316	0.062	1E-7	0.089 (age, age^2^, sex, age*sex age^2^*sex, current smoking)
3a	− 510.396	0.251 (0.090)	0.082 (0.055)	1E-3 (4E-2)	0.091
3b	− 508.105	0.359 (0.256)	0.074 (0.087)	1E-8 (3E-3)	0.089
**cg17058475 at** ***CPT1A***					
0	− 692.475	0.302	0.069	4E-7	–
1	− 668.034	0.365	0.071	4E-9	0.038
2a	− 665.591	0.356	0.071	9E-9	0.042
2b	− 662.173	0.351	0.071	1E-8	0.051
1–2 (Reduced)	− 666.050	0.355	0.071	8E-9	0.043 (age, current smoking)
3a	−665.471	0.298 (0.062)	0.092 (0.060)	8E-4 (1E-1)	0.044
3b	NC				
**cg18181703 in** ***SOCS3***					
0	− 555.561	0.486	0.063	8E-18	–
1	− 518.163	0.557	0.063	2E-21	0.055
2a	− 517.421	0.551	0.064	4E-21	0.058
2b	−514.324	0.559	0.063	1E-21	0.062
1–2 (Reduced)	− 515.994	0.566	0.063	3E-22	0.057 (age, age^2^, center, current and former smoking)
3a	−515.889	0.553 (0.020)	0.071 (0.045)	1E-11 (3E-1)	0.062
3b	−515.663	0.585 (0.085)	0.068 (0.104)	2E-22 (2E-1)	0.060
**cg06500161 in** ***ABCG1***					
0	− 195.927	0.323	0.070	3E-8	–
1	−181.366	0.330	0.069	1E-8	0.028
2a	−177.208	0.313	0.069	1E-7	0.039
2b	−176.433	0.305	0.069	1E-7	0.041
1–2 (Reduced)	− 184.146	0.306	0.069	1E-7	0.026 (sex, center)
3b	NC				
3c	NC				

*Abbreviations: c*^*2*^ Household variance component, *h*^*2*^ heritability variance component, *NC* nonconvergence of the household variance component model(s), *Prop Var Exp by Covar* proportion of variance explained by covariates, *SNP* single nucleotide polymorphisms

^a^The fixed covariates introduced in a stepwise fashion across Models 1 (age, age^2^, sex, age*sex, age^2^*sex), 2a (center), and 2b (current and former smoking, indicator variables) were then screened at *p* < 0.1, to yield a reduced model. Then in Models 3a (‘early life’ shared environment, 647 siblings or half-siblings, within 15 years of each other in 255 households) and 3b (‘later life’ shared environment; 128 parents, or in the case of multiple pairings those with the youngest offspring, in 64 households) variance components were separately introduced individually to this reduced model

For the two *CPT1A* CpG sites*,* which were correlated at 0.74 in our data*,* 1 distant (±250–500 kb) variant, rs17601808, was significantly associated with cg00574958 (*P* value = 0.01) and cg17058475 heritability estimates (*P* value = 5E-3) after accounting for fixed effects from the reduced model (see Table 1), resulting in significant but attenuated heritability estimates (h^2^ = 0.24, *P* value = 5E-4, VE = 11.8%; h^2^ = 0.31, *P* value = 2E-5, VE = 6.2%; respectively). For the *SOCS3* site, one local (±250 kb) genetic variant, rs7220979, and two distant variants, rs9908993 and rs17736494, were significantly associated with cg18181703 heritability after accounting for fixed effects (*P* values ≤0.05), resulting in similar heritability estimates (h^2^ = 0.58, *P* value = 1E-14; VE = 7.7%) as in previous models. Including these 3 SNPs resulted in nonsignificant estimates for center and former smoking, and dropping these nonsignificant fixed covariates also yielded similar heritability estimates (h^2^ = 0.57, *P* value = 1E-14; VE = 7.4%). At the *ABCG1* site*,* 2 local (rs220245 and rs225434, *P* values *=* 0.03 and to 3E-4) and 1 distant genetic variant (rs8128650, *P* value *=* 0.04) were associated with cg06500161 heritability (h^2^ = 0.32, *P* value *=* 5E-5, VE = 6.8%) after accounting for fixed effects.

### Familial correlations

**Fig. 2:**
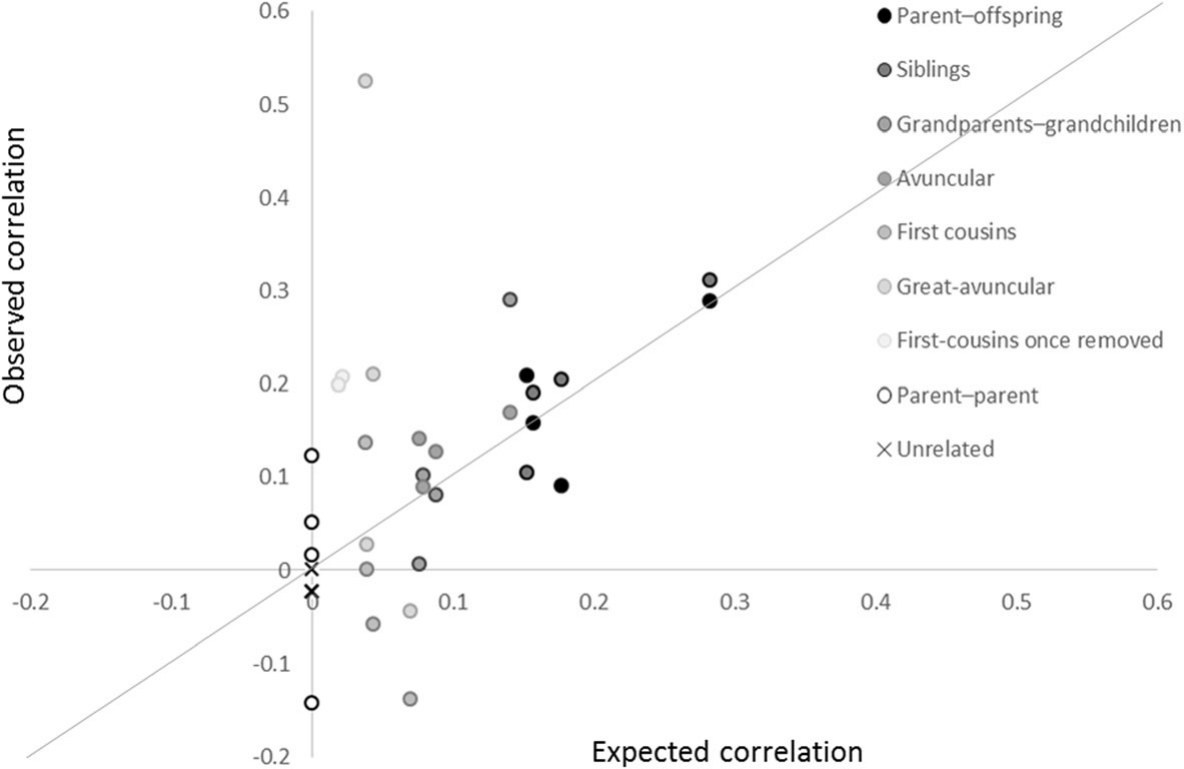
Four CpG methylation sites for metabolic syndrome and their expected and observed correlations of relative pairs after accounting for age, age^2^, sex, center, and current smoking showing clustering along the line of unity (in black)

We then estimated familial coefficients across a number of relationship pairings, before and after creating a residual adjusting for age, age^2^, sex, center, and current smoking, which were retained in more than 1 reduced heritability model (see Table 1). The use of residuals to account for these fixed covariates generally decreased the estimates slightly (Table 2). We also observed that strata informed by more relative pairs (eg, parent–offspring, sibling and avuncular) exhibited correlations closer to our expectation based on relatedness and the observed heritability of the specific CpG site (see Fig. 2). For example, for cg18181703 in *SOCS3* the correlations estimated for each of these relative pairs as well as grandparent–grandchildren were nominally significant (*P* value < 0.05), and were 0.01 to 0.15 greater than our expected correlation.

**Table 2:** Variation in estimated intra- and interclass correlation coefficients across relative paired groups and subtypes (*N* > 50) at 3 MetS-related CpG methylation loci unadjusted and adjusted for fixed covariates in 993 participants from 188 families with nonmissing covariates and methylation information at visit 2 in the GOLDN study

Pair Type	N^a^	Familial Correlations								
		Expectation^b^	cg00574958 at *CPT1A*		cg17058475 at *CPT1A*		cg18181703 in *SOCS3*		cg06500161 in *ABCG1*	
			h^2^ = 0.316		h^2^ = 0.355		h^2^ = 0.566		h^2^ = 0.306	
			Unadj.	Adj.	Unadj.	Adj.	Unadj.	Adj.	Unadj.	Adj.
Parent–offspring	541	*h*^*2*^/2	**0.1721**	**0.1578**	0.0986	0.0900	**0.2964**	**0.2887**	**0.2267**	**0.2081**
Mother–daughter	158	*h*^*2*^/2	**0.2240**	**0.2565**	**0.2094**	**0.2572**	**0.2590**	**0.2591**	**0.1755**	0.1537
Mother–son	146	*h*^*2*^/2	**0.2302**	0.1868	0.0750	0.0625	**0.2207**	**0.2525**	**0.1775**	**0.1766**
Father–daughter	129	*h*^*2*^/2	0.0422	0.0729	0.1766	0.1774	**0.4510**	**0.4239**	**0.3417**	**0.3415**
Father–son	108	*h*^*2*^/2	0.1967	**0.2588**	0.0480	0.0172	**0.2813**	**0.2477**	**0.2071**	**0.1982**
Siblings	588	*h*^*2*^/2	**0.2224**	**0.1906**	**0.2071**	**0.2043**	**0.3295**	**0.3114**	**0.1185**	**0.1039**
Brother–brother	145	*h*^*2*^/2	**0.2396**	**0.2333**	**0.2328**	**0.2359**	**0.2668**	**0.2413**	0.0647	0.0555
Sister–brother	276	*h*^*2*^/2	**0.2075**	**0.1562**	**0.1496**	**0.1556**	**0.3252**	**0.2774**	0.1187	0.0978
Sister–sister	167	*h*^*2*^/2	**0.2245**	**0.3141**	**0.2680**	**0.2517**	**0.3769**	**0.3577**	0.1490	0.1478
Grandparents–grandchildren	75	*h*^*2*^/4	0.0924	0.1020	0.0781	0.0807	**0.3342**	**0.2892**	0.0164	0.0064
Avuncular	553	*h*^*2*^/4	0.0685	0.0893	**0.1323**	**0.1272**	0.1440	**0.1691**	**0.1632**	**0.1410**
First cousins	247	*h*^*2*^/8	0.0227	0.0007	− 0.0393	− 0.0584	− 0.1394	− 0.1392	0.1411	0.1362
Great-avuncular	53	*h*^*2*^/8	0.0005	0.0266	0.1279	0.2092	− 0.0215	− 0.0453	**0.5514**	**0.5249**
First cousins once removed	71	*h*^*2*^/16	0.0486	0.1987	0.3085	0.2066	− 0.2466	− 0.2190	0.2418	0.1984
Parent–parent	65	0	0.1412	0.1221	− 0.0982	− 0.1432	0.0507	0.0164	0.0613	0.0505
Unrelated^c^	91	0	− 0.0089	− 0.0236	0.0126	− 0.0231	− 0.0181	− 0.0235	− 0.0011	− 0.0002
MS Concordant	91	0	0.1420	0.1130	0.1602	0.1270	− 0.0399	− 0.0580	− 0.0051	− 0.0408
MS Discordant	76	0	− 0.1354	− 0.1284	− 0.1547	− 0.1515	− 0.1718	− 0.1566	0.0291	0.1017

Values in bold represent estimates that are nonzero with a *P* value < 0.05

*Abbreviations: Adj.* Calculated on residuals created after adjusting for fixed covariates age, age^2^, sex, center, and current smoking, *MS* National Cholesterol Education Program Expert Panel on Detection, Evaluation, and Treatment of High Blood Cholesterol in Adults Metabolic Syndrome status at visit 2, *Unadj.* unadjusted for any covariates

^a^Pairings may not be independent

^b^The expected correlation under a genetic model with a heritability of h^2^

^c^Overall unrelated correlation assigned by subsetting to the 182 individuals from distinct families and randomly pairing them, whereas concordant and discordant strata were calculated after randomly pairing within or across the National Cholesterol Education Program Expert Panel on Detection, Evaluation, and Treatment of High Blood Cholesterol in Adults criteria for MetS cases (*N* = 76) and controls (*N* = 106)

Although not statistically significantly different from other pairings (heterogeneity *P* value ≥0.3), the correlations estimated for sister pairs were the largest across all sites (see Table 2). We observed nonsignificant (*P* values ≥0.4) positive correlations at 3 CpG sites among parent pairs (65 independent pairs), which were between 0.02 and 0.12 greater than expected. Among unrelated pairs, we observed correlations that were closer to our expectation of no correlation (eg, all within 0.02 of zero), which supports the upward bias of shared household environments on familial correlations. When we further paired this unrelated with respect to MetS status, the correlation at the 2 *CPT1A* CpG sites were biased upwards among concordant pairs, and downwards from the null among discordant pairs.

## Discussion

Although several animal models have established the transgenerational epigenetic inheritance of metabolic diseases, substantial hurdles remain to describing the inheritance of DNA methylation in humans [4]. This is partly because of the currently limited availability of large multigenerational or family-based studies with CpG methylation data and other relevant social and environmental factors. Previous studies found that the methylome-wide heritability patterns reflect negligible heritability at most CpG sites, and that some CpG sites (14–80%) are regulated, in part, by local genetic variation [7, 13, 14]. Only one previous study has also tried to portion the variance caused by shared environmental factors as a means of better understanding how methylation may be inherited across generations, concluding that shared environments, captured by nuclear family membership, contribute little to the observed methylome heritability [13]. In contrast, our overall findings support roles for both shared environment and genetic variation in explaining the heritability at the 4 CpG sites in 3 methylation loci previously associated with MetS or several of its subcomponents that we considered.

We observed an improvement of our MetS heritability estimates after including CpG sites, which is consistent with the transgenerational epigenetic inheritance as a contributor to the missing heritability in complex traits like MetS.

We found that CpG site heritability estimates generally increased as additional fixed effects for environmental and genetic covariates were added to the variance component model, but that the heritability estimates were statistically indistinguishable. Although including random effects of early or late life shared environments also did not markedly change CpG heritability estimates, we were able to identify a measurable, and at times significant influence of shared environment on MetS and CpG site heritability, which affirms the joint role of both shared environmental and genetic influences on MetS and related methylation. These observations collectively point to the methodologic importance of including shared environmental factors, especially in childhood or adolescence, when modeling heritability estimates at later time points.

Additionally, we estimated familial correlations (with and without adjustments for key covariates) across various types of relative pairs. We observed that correlations generally followed our expectation based on relatedness, but in the case of sister and parent pairs we observed nonsignificant trends toward greater correlation than expected. We posit that shared social and environmental factors may make particular relative pairs appear more similar than we would expect based on their relatedness alone, which could lead to further inflation of heritability and familial correlation estimates.

## Conclusions

Previous research has not been able to address the extent of inflation of epigenetic inheritance estimates by shared environmental effects, even though the sharing of social or environmental exposures within households may be a key driver of the observed similarity of methylation profiles within families [7, 13, 14]. Our results indicate that MetS CpG site heritability is extremely robust, even though both shared environmental and genetic influences play roles in the intergenerational patterning at these sites. Although the current analysis brings us a step closer to deciphering the complex action of transgenerational epigenetic inheritance, shared environments, and genetic variation in DNA methylation profiles in humans, without much larger families including 3 or more generations or richer data on life course environmental risk factors, we are unable to fully decompose the role of each actor at the CpG sites for MetS considered here. Yet, this study does outline an interesting and a flexible statistical framework for testing such models in the context of human family studies. Future work should consider these, and other methods, to replicate our heritability and familial correlation findings to further describe the mechanisms of epigenetic inheritance in human populations.

## Abbreviations

CpG: Cytosine-phosphate-Guanine
GAW20: Genetic Analysis Workshop 20
GOLDN: Genetics of Lipid Lowering Drugs and Diet Network
MetS: Metabolic syndrome
SE: Standard error
SNP: Single nucleotide polymorphism
VE: Variance explained
